# ACKR3 Upregulation Under Hyperglycemia Associates with Endothelial Dysfunction

**DOI:** 10.34172/apb.46517

**Published:** 2026-05-10

**Authors:** Seyedeh Zohreh Azarshin, Zahra Abedi Kichi, Shahram Rabbani, Mohammad Ali Boroumand, Mousa Golalizadeh Lahi, Mehrdad Behmanesh

**Affiliations:** ^1^Department of Genetics, Faculty of Biological Sciences, Tarbiat Modares University, Tehran, Iran; ^2^Institute for Cardiovascular Prevention (IPEK), Ludwig-Maximilians University Munich, 80336 Munich, Germany; ^3^Research Center for Advanced Technologies in Cardiovascular Medicine, Tehran University of Medical Sciences, Tehran, Iran; ^4^Cardiovascular Diseases Research Institute, Tehran Heart Center, Tehran University of Medical Sciences, Tehran, Iran; ^5^Department of Statistics, Faculty of Mathematical Sciences, Tarbiat Modarres University, Tehran, Iran

**Keywords:** ACKR3, Diabetes mellitus, Endothelial cell, GPCRs, Hyperglycemia

## Abstract

**Purpose:**

Diabetes is a metabolic disorder characterized by chronic hyperglycemia and is associated with cardiovascular complications. Endothelial cell dysfunction is a common feature of both diabetes and its vascular complications. In this study, RNA-seq data from aortic specimens of diabetic patients were analyzed to identify candidate genes that are potentially involved in regulating endothelial cell function under hyperglycemic conditions. Among the identified GPCRs, *ACKR3* was singled out as a promising candidate.

**Methods:**

Expression of *ACKR3* in the vessels of diabetic animals and the HUVEC cell line under hyperglycemia conditions was assessed. Subsequently, the function of the *ACKR3* gene was investigated by downregulation of ACKR3 at hyperglycemic conditions in the HUVEC cell line.

**Results:**

Our data showed that expression of Ackr3 in the vessels of diabetic animals was increased. Furthermore, treatment of HUVECs under hyperglycemic conditions (25 mM D-glucose) for 24 h and 48 h resulted in elevated ACKR3 expression at both mRNA and protein levels. Functional analyses revealed that ACKR3 downregulation resulted in significant reductions in oxidative stress levels, inflammation, and the rate of endothelial cell apoptosis under hyperglycemic conditions. Additionally, ACKR3 downregulation reduced the expression of adhesion molecules (ICAM and VCAM), leading to decreased monocyte adhesion under hyperglycemic conditions.

**Conclusion:**

Our findings suggest that hyperglycemia-induced ACKR3 up-regulation may contribute to endothelial cell dysfunction in diabetic vessels. Therefore, targeting ACKR3 could offer valuable insights for developing therapeutic strategies against diabetes-related vascular complications.

## Introduction

 Diabetes mellitus (DM) is a common metabolic disease indicated by high levels of blood glucose due to defective insulin secretion or resistance to insulin.^1^ The World Health Organization (WHO) calculated that diabetes may be the 7th leading cause of death, and the prevalence of diabetes is estimated to increase by more than 600 million by 2030.^2^* Chronic hyperglycemia leads to *severe complications such as cardiovascular diseases (CVDs), neuropathy, kidney damage, and several other problems.^3^ Diabetes significantly enhances the Incidence of atherosclerosis, and 50%-80% of deaths among patients with diabetes are due to its cardiovascular complications.^2^

 Endothelial dysfunction is the key early event in the pathogenesis of cardiovascular complications of diabetes, driven primarily by chronic hyperglycemia. Endothelial dysfunction is considered an early stage of atherosclerosis.^4^ Several studies have linked endothelial dysfunction and atherosclerosis with insulin resistance, diabetes, obesity, and other inflammatory factors.^5^ In diabetic patients, endothelial dysfunction is caused by multiple factors, including high glucose levels, inflammation, oxidative stress, and insulin resistance, with mediators including nitric oxide (NO), reactive oxygen species (ROS), AGEs, and inflammatory cytokines such as TNF-α and IL-6, which play crucial roles in exacerbating vascular damage.^6,7^ Among these, oxidative stress and inflammation are important causes of endothelial dysfunction following hyperglycemia.^3^ ROS generation contributes to endothelial dysfunction through multiple signaling pathways, including membrane lipid peroxidation and the activation of NF-κB, G-proteins, and ion channels.^8,9^ Subsequently, following an increased level of inflammation under hyperglycemia, endothelial dysfunction manifests as enhanced expression levels of adhesion molecules such as ICAM and VCAM, changes in vascular permeability, and finally, increased binding of leukocytes to endothelial cells, all of which are important events in the progression of vascular complications of diabetes.^10^

 Inflammatory mediators activate cell signaling pathways through receptors such as G-protein-coupled receptors (GPCRs).^11^ GPCRs constitute the superfamily of transmembrane receptors that transduce extracellular signals into critical biological processes through the activation of intermediary G proteins or β-arrestin. The GPCRs superfamily can recognize various endogenous ligands, such as hormones, neurotransmitters, chemokines, peptides, and extracellular calcium. They regulate a wide variety of biological processes, such as cell‒cell interactions, inflammation, and energy homeostasis.^12^ Given that GPCRs have been implicated in many metabolic disorders, they are currently the best target for therapeutic drugs.­^12^ Several previous studies have shown that GPCRs are differentially expressed in diabetes and vascular complications.^13–16^ Currently, more FDA-approved drugs targeting GPCRs are still being develop. ^17^

 The present study aims to evaluate the expression and function of one of the GPCRs potentially involved in the development of diabetic vascular complications linked to endothelial dysfunction. We analyzed RNA-seq data derived from the aortic endothelial cells of patients with diabetes to identify GPCRs with therapeutic relevance. Among these, the *ACKR3* gene was selected as a key diabetes-related GPCR, and its function in endothelial cells under hyperglycemic conditions was subsequently investigated.

## Materials and Methods

###  Bioinformatics Analysis

 RNAseq (GSE77108) data analysis of aortic endothelial cells from diabetic patients and healthy individuals (n = 3 per group) was performed to identify GPCRs with significant differential expression with adjusted *P*-values (padj) < 0.05 (Supplementary File 1, Table S1). In the next step, GWASs were conducted to identify GPCRs that have genetic variants related to diabetes and its cardiovascular complications. Moreover, the GSE267930 dataset was analyzed to confirm the expression changes of GPCRs following induction of dysfunction in endothelial cells (n = 3 per group) (Supplementary File 1, Table S1). Among all candidate GPCRs, we focused on those located in the chromosomal hotspot region associated with diabetes (2q37) and linked to cardiovascular complications of diabetes. Finally, the *ACKR3* gene was selected for further studies. The associations of *ACKR*3 with other genes and cellular pathways were predicted via the JASPAR (9th release, V4.0, 2022) ^18^، KEGG (V 103.0, July 2022, Kanehisa Laboratories) ^19^ Harmonizome (2022 Database, Oxford). ^20^

###  Experimental Models of Diabetes

 In the present study, to induce diabetic animal models and avoid potential confounding effects of hormonal fluctuations associated with the estrous cycle in female rats, which could influence glucose metabolism and diabetes outcomes, we obtained 16 male Wistar rats weighing between 200 ± and 20 grams from the Pasteur Institute of Iran. The rats were housed in a temperature-controlled room with a 12-h light‒dark cycle and free access to food and water. Our research was performed according to the ARRIVE guidelines, for the Care and Use of Laboratory Animals^21^ (“Ethics Committee of Tarbiat Modares University”; IR.MODARES.REC.1399.192), and all experiments were performed in accordance with relevant guidelines and regulations. Following a one-week acclimatization period, the rats were randomly assigned to either the control or experimental groups. Diabetes was induced in the experimental group via a single intraperitoneal injection of streptozotocin (STZ) at a dose of 55 mg/kg, dissolved in 0.01 M sodium citrate buffer (pH 4.0). The control group received an equivalent volume of sodium citrate buffer. The levels of blood glucose were measured three days after STZ injection to confirm diabetes induction. Moreover, to minimize bias, researchers performing blood glucose measurements and tissue analysis were blinded to the group assignments. Rats with blood glucose levels above 250 mg/dL were classified as diabetic. After 28 days of diabetes induction^22^, the rats were anesthetized with intraperitoneal injections of ketamine (100 mg/kg) and xylazine (10 mg/kg), and finally, euthanasia was performed by overdose of sodium thiopental (150 mg/kg) according to AVMA guidelines 2020 edition. Subsequently, tissues were harvested for further experiments. Inclusion criteria included male Wistar rats weighing 200 ± 20 grams with no signs of illness or distress during the acclimatization period. Exclusion criteria were applied to rats that did not achieve blood glucose levels > 250 mg/dL three days post-STZ injection. Additionally, humane endpoints were established to ensure animal welfare. Rats exhibiting severe weight loss, persistent hypoglycemia, or signs of extreme distress were euthanized early according to AVMA guidelines. Also, STZ-induced diabetic rats with a level of blood glucose greater than 600 mg/dL were excluded from the experiment.

###  Cell Culture and Treatments

 To investigate the effects of hyperglycemia on *ACKR3* gene expression in endothelial cells, human umbilical vein endothelial cells (HUVECs, Pasteur Institute, Iran) were used. HUVECs were cultured in Dulbecco’s modified Eagle’s medium (DMEM) (Gibco) supplemented with 10% (v/v) fetal bovine serum (FBS, Gibco) and 1% penicillin‒streptomycin (100 U/ml penicillin and 100 μg/ml streptomycin) at 37°C in a humidified atmosphere containing 5% CO2. To mimic a condition close to normoglycemic (NG) and hyperglycemic (HG), HUVECs were treated with media containing 5 mM or 25 mM D-glucose, respectively, for 24 and 48 hours. For the monocyte adhesion assay, THP1 cells were cultured in Roswell Park Memorial Institute 1640 medium (RPMI 1640, 11530586, Gibco, Thermo Fisher Scientific) supplemented with 10% FBS, 1% penicillin/streptomycin, and 2 mM L-glutamine.

###  Gene Expression Analysis via Quantitative PCR 

 Total RNA was extracted from the HUVECs, heart tissue, and vessels using RiboEX reagent (GeneAll, Korea) according to the manufacturer’s instructions. The quality and quantity of the RNA were confirmed via electrophoresis and spectrophotometry. Subsequently, 3-5 μg of total RNA was reverse transcribed into cDNA via a cDNA reverse transcription kit (TAKARA, Japan) according to the manufacturer’s recommendation. Gene expression analysis was performed by a stepOne^TM^ PCR system (Applied Biosystems/MDS SCIEX, Foster City, CA, USA) using 5X EvaGreen I master mix (Solis BioDyne, Estonia). Each sample had at least three independent biological and technical replicates. The relative expression levels of target mRNAs were normalized to the* β-actin *gene, and differential gene expression analysis was evaluated via the 2^-ΔΔCT^ method.^23^ sequences of primers that were used in our study are listed in Supplementary File 1, Table S2.

###  ACKR3 Gene Knockdown

 The expression of the *ACKR3* gene was reduced using a specifically designed DNAzyme. DNAzyme design with the 5ʹ-GGATGGTGAGGAGGCTAGCTACAACGAAACCCACAGG-3ʹ sequence was performed by Oligo 7 (DBA Oligo, USA), Snap Gene (GSL Biotech LLC, USA) software, and its specificity was confirmed by NCBI-Blast. HUVECs were cultured under HG or NG conditions for 24 and 48 hours, then were transfected with either ACKR3 DNAzyme ^24^ or scrambled DNAzyme via the Lipofectamine 2000 reagent (Invitrogen Life Technologies, USA). The *ACKR3* downregulation efficiency was confirmed by qPCR. All the data represent at least three independent biological replicates.

###  Enzyme-Linked Immunosorbent Assays

 To assess the effect of ACKR3 on regulating IL-6 cytokine secretion under hyperglycemia conditions, HUVECs were exposed to NG or HG conditions for 24 and 48h. Subsequently, cells were transfected with either scrambled DNAzyme or ACKR3 DNAzyme. After 48h post-transfection, the levels of IL-6 were measured in the supernatant media of HUVECs using an enzyme-linked immunosorbent (ELISA) assay.

###  Reactive Oxygen Species (ROS) Assay

 To assess the regulatory function of the ACKR3 in the production of intracellular ROS, the ROS production was measured via the OxiSelect Intracellular ROS Assay Kit (STA-342, Cell Biolabs, San Diego, CA, USA) according to the manufacturer’s instructions. For this purpose, after 24 h and 48 h treatment with normal or hyperglycemia conditions, cells were transfected with the ACKR3 DNAzyme or scrambled DNAzyme to downregulate *ACKR3 *expression. At the final stage of the experiment, the cells were stained with *2 ′,7 ′ -*dichlorodihydrofluorescein diacetate (DCFH-DA). The excess DCFHDA dye was removed via washing cells three times with PBS. Fluorescence intensity, indicative of ROS levels, was measured at excitation/emission wavelengths of 485/530 nm using fluorescence spectrophotometry (BioTek citation 3 cell imaging, Agilent). All the data represent at least three independent biological replicates and 2 technical replicates.

###  Mitochondrial Isolation Method

 To assess the effect of ACKR3 on the level of ROS generation in mitochondria, mitochondrial isolation was performed using the Mitochondria Isolation Kit for Mammalian Cells (Thermo Scientific, cat. number: 89874) according to the manufacturer’s recommendation. For this purpose, cell treatment and transfection were performed as mentioned above. Finally, mitochondrial ROS generation was assessed based on previous research. ^25,26^

###  CFSE Proliferation Assay

 To evaluate the impact of *ACKR3* downregulation under HG and NG conditions on endothelial cell proliferation, HUVECs were labeled with carboxy fluorescein succinimidyl ester (CFSE, Promega) according to the manufacturer’s instructions. Then, HUVECs were seeded and treated with NG or HG conditions for 24h and 48h. At 48h after transfection of the cells according to the method described, the proliferation of CFSE-labeled HUVECs was assessed via flow cytometry and compared with that of control cells. All the data were from at least three biological replicates and 2 technical replicates.

###  Apoptosis and Cell Cycle Assay

 To assess the effect of ACKR3 on cell apoptosis, Annexin-V propidium iodide (PI) staining was performed. Briefly, HUVECs were exposed to NG or HG for 24h or 48h, and *ACKR3* downregulation was conducted as previously described. HUVECs were trypsinized with Trypsin-EDTA 0.25% and labeled with the Annexin-V FITC Apoptosis Detection Kit (Sigma) according to the manufacturer’s instructions. The degree of cell apoptosis was evaluated via flow cytometry. For cell cycle analysis, HUVECs were seeded in a 24-well plate at a density of 10^4^ cells/well. Cell treatment and transfection were then performed as described above at 24h and 48h. After transfection, HUVECs were harvested and fixed in 70% ethanol. Subsequently, cells were labeled with propidium iodide (PI; Sigma) and incubated for 30 min at room temperature. Finally, to assess the cellular DNA content, all the samples were analyzed via BD Biosciences flow cytometry. All the data presented in this study were obtained from 3 biological replicates.

###  Monocyte Adhesion Assay Using Red CMTPX

 HUVECs were cultured on gelatin-coated 96-well plates and treated with NG or HG conditions for 48h. ACKR3 downregulation was then performed as previously described. Finally, the THP-1 cells were labeled with red CMTPX for 60 min, following the manufacturer’s guidelines (C34552, CellTracker^TM^ Red CMTPX Dye, Invitrogen^TM^). Confluent monolayers of HUVECs were cocultured with labeled THP-1 cells for 30 min at 37°C. Then, the monolayer of cells was washed three times with PBS to remove nonadherent THP-1 cells. Representative images were captured using a live-cell inverted microscope (Olympus Germany) equipped with a fluorescence and bright-field camera. Fluorescent light of adherent THP-1 to HUVECs was measured by excitation/emission (577 nm/ 602 nm) using fluorescence spectrophotometry (BioTek citation 3 cell imaging, Agilent).

###  Senescence-Associated β-Galactosidase (SAβ-Gal) Staining

 The impact of ACKR3 downregulation on endothelial cell senescence was investigated via a SAβ-Gal staining assay as previously described.^27^ Briefly, at the end of the experiment, fixations of HUVECs were performed with 4% paraformaldehyde for 10 min at room temperature. The cells were subsequently incubated with fresh X-gal solution (5-bromo-4-chloro-3-indolyl-β-D-galactopyranoside (1 mg/ml), potassium ferrocyanide (5 mM), potassium ferricyanide (5 mM), and MgCl2 (2 mM)) overnight at 37°C. Finally, to evaluate senescent endothelial cells, blue/green-stained ECs were quantified via an inverted microscope.

###  Western Blot Analysis

 To evaluate the expression change of candidate proteins, total proteins were extracted from HUVECs via radioimmunoprecipitation (RIPA) lysis buffer comprising protease and phosphatase inhibitor cocktails (complete Mini Tablets and PhosSTOP, Roche, Switzerland). The concentration of extracted protein was measured via the Bradford assay at 490 nm. Equal amounts of protein (20 μg) were separated on 10% precast polyacrylamide gels via SDS-PAGE and subsequently transferred to polyvinylidene fluoride (PVDF membrane), and 5% BSA was used for membrane blocking. The membranes were incubated with the primary antibody at 4°C overnight. After washing, the incubation of membranes with secondary horseradish peroxidase (HRP)-conjugated antibodies was performed. The specific protein bands were subjected to chemiluminescence by an enhanced chemiluminescence (ECL) kit (Amersham, UK). For all the samples, β-ACTIN was used as an internal control. All the bands were quantified via ImageJ (RRID: SCR_003070). All antibodies Cat. No that used for this study are reported in Supplementary File 1, Table S3.

###  Catalase and Superoxide Dismutase Activity

 To investigate the role of ACKR3 downregulation under hyperglycemia conditions, HUVECs were transfected with ACKR3 DNAzyme or Scr-DNAzyme after treatment with NG or HG conditions. At the end of the experiments, the cells were trypsinized, and catalase and SOD activity were measured via Koushan Zist kits (Iran), following the standard protocols. The activity of the Catalase enzyme was investigated by measuring the amount of formaldehyde at a wavelength of 540 nm. The activity of the Superoxide Dismutase enzyme was measured at a wavelength of 460 nm, relative to a blank control based on inhibition of the formation of formazan.

###  Statistical Analysis

 Statistical analysis was conducted via GraphPad Prism software (version 9). The data are presented as the means ± standard deviations (SDs) (n = 3 independent experiments). Statistical significance between the two groups was compared via Student’s t-test. Moreover, statistical significance among three or more groups was analyzed via one-way ANOVA followed by Tukey’s HSD post hoc test for multiple comparisons. Normality and homogeneity of variance were assessed using Shapiro-Wilk and Levene’s tests, respectively. In cases where parametric assumptions were violated, Welch’s ANOVA was performed to confirm robustness. Effect sizes were reported as omega-squared (ω^2^) with 95% confidence intervals using the effect size. A P value of < 0.05 was considered statistically significant. Moreover, to address multiplicity, *P*-values were adjusted using the Benjamini–Hochberg false discovery rate (FDR) procedure.

## Results

###  Identification of the GPCR Involved in Diabetes and Endothelial Cell Dysfunction

 To identify the GPCR that has an impact on endothelial cell dysfunction, RNA-seq data from the aorta samples of diabetic patients and healthy individuals were analyzed. We search for GPCRs that are associated with diabetes or its complications using GWAS. Expression change of these GPCRs in endothelial cells of diabetic patients revealed that 5 GPCRs *(ACKR3, GRM7, CALCRL, GPR160, EDNRA) *showed significantly different expression. RNAseq analysis of the dataset related to induced endothelial dysfunction showed that *ACKR3* and *CALCRL* are differentially expressed. Finally, we selected the *ACKR3* gene by far the strongest statistical evidence of dysregulation in induced endothelial dysfunction (FDR = 4.13 × 10⁻⁵³ vs. 3.80 × 10⁻⁴ for CALCRL). Moreover, this gene is located at 2q37.3. The selection of 2q37 as a hotspot is supported by multiple genome-wide linkage and association studies, indicating its strong linkage to type 2 diabetes susceptibility. The well-studied *CAPN10* gene, located within 2q37, further confirms this locus as a hotspot for diabetes susceptibility. The haplotype present at this chromosomal locus can be associated with susceptibility to diabetes. Notably, this chromosomal region harbors several long-arm GPCR genes where variants have been reported to associate with increased diabetes risk, especially in certain populations such as Mexican-Americans.^28–30^ (Figure 1).

**Figure 1:**
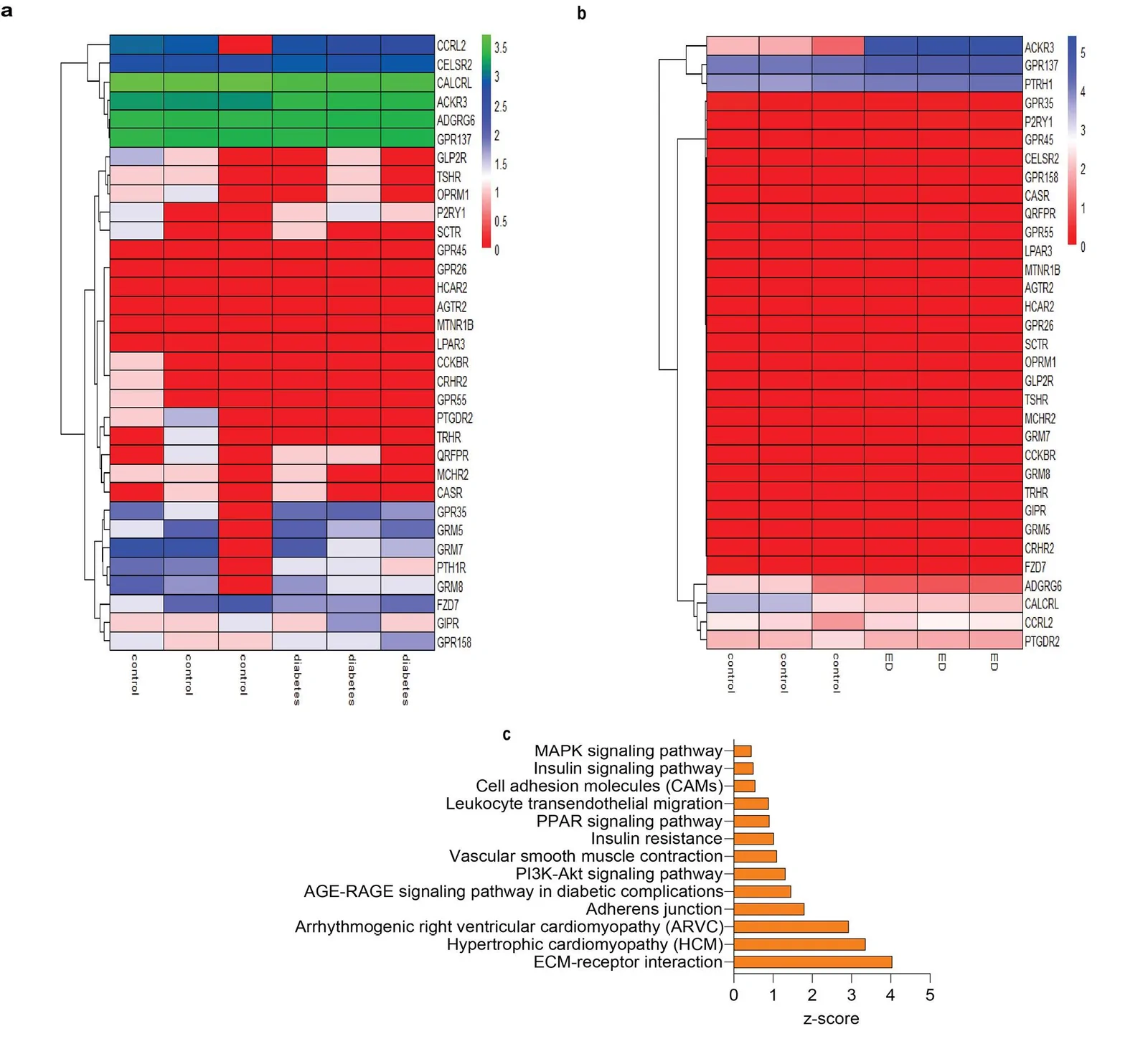
The *ACKR3* gene was selected based on bioinformatics analysis. The available GEO datasets (GSE77108 & GSE267930) were analyzed, and a relative heatmap of GPCRs was drawn. a: Heatmap of differentially expressed gene (DEG) GPCRs from an RNA-seq study obtained from the aortic endothelial cells of diabetic patients (n = 3) compared with those of healthy individuals (n = 3). b: Heatmap of differentially expressed GPCRs in control endothelial cells and endothelial dysfunction-induced cells. c: The ACKR3 is related to several important pathways related to diabetes complications

###  Hyperglycemia Increased the Expression of ACKR3 in the Vessels of Diabetic Rats & Huvecs

 The STZ-induced diabetic rat model is one of the most reliable animal models for investigating gene expression changes following hyperglycemia induction.^31^ Therefore, we utilized this model to investigate *Ackr3* expression following hyperglycemia induction. To confirm the induction of diabetes, blood glucose was measured three days after STZ injection. Rats with blood glucose levels higher than 250 mg/dl were considered diabetic rat models. Furthermore, to confirm the sustained induction of diabetes, blood glucose levels in STZ-induced diabetic rats were monitored throughout the study (Supplementary File 1, Figure S1). Moreover, STZ-induced diabetic rats with a level of blood glucose greater than 600 mg/dL were excluded from the experiment. Finally, we have 5 rats in each group. According to a previous study, the onset of endothelial dysfunction in STZ-induced diabetic models occurs 28 days after the induction of diabetes.^22^ The *Ackr3* expression was upregulated in the vessels of diabetic rats (*P*-value: 0.002). In contrast, *Ackr3* expression was decreased in the ventricles and atria of diabetic rats (Figure 2 a-e). To assess the functional role of the *ACKR3* gene during hyperglycemia, HUVECs were treated with 5 mM glucose (NG, normal glucose) or 25 mM glucose (HG, high glucose) for 24 or 48 hours. Treatment of HUVECs with HG increased ACKR3 expression at both the mRNA and protein levels by approximately 3 and 1.8-fold, respectively (Figure 2 f-h).

**Figure 2:**
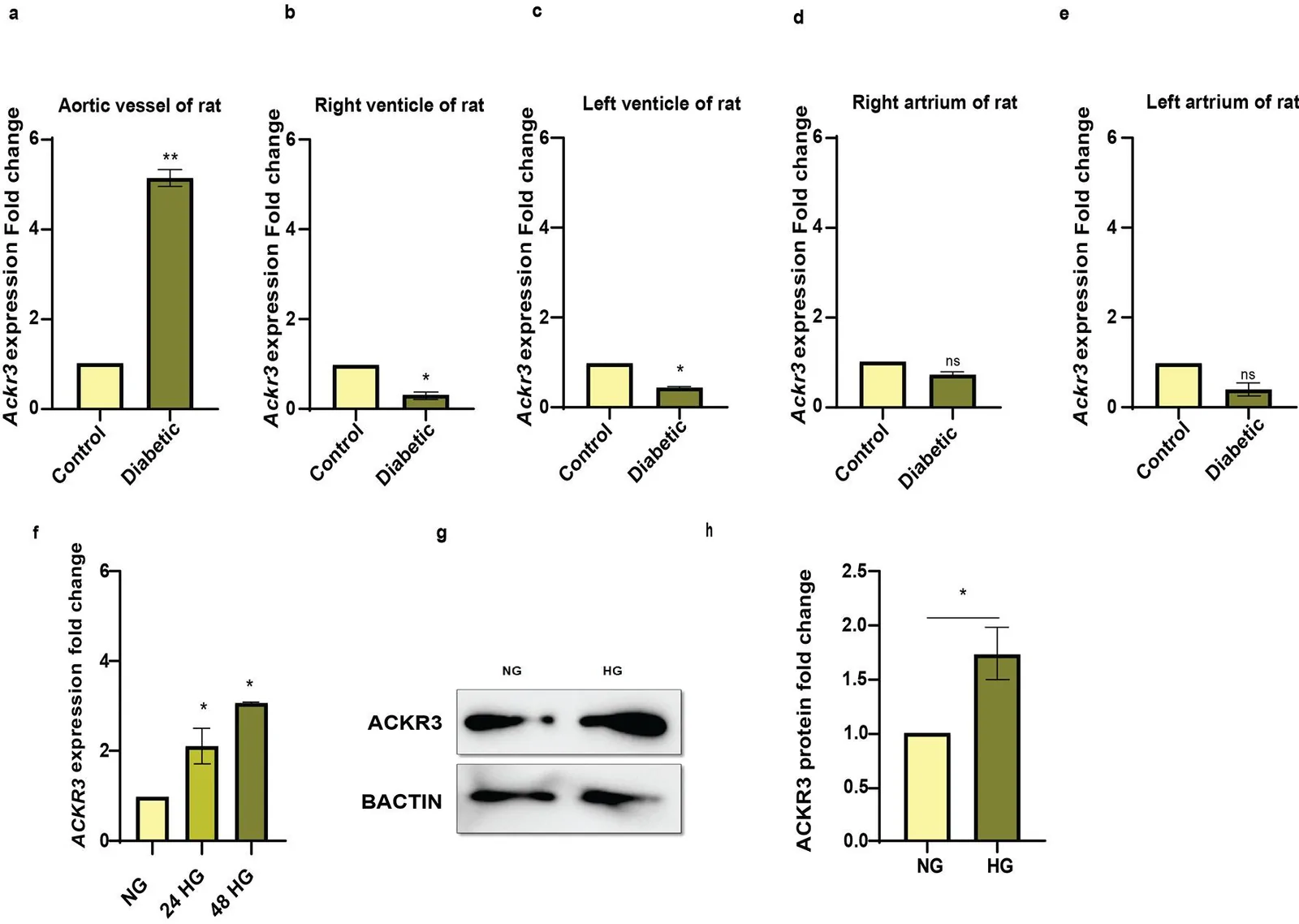
Hyperglycemia significantly increased the expression of ACKR3 in rat vessels. *Ackr3* expression in the cardiovascular tissue of STZ-induced diabetic rats (n = 5) and control rats (n = 5) (a-e); Student’s t-test, a: ** p: 0.002,η2: 0.9962, 95% CI(3.350 to 4.892). b: * *P*-value: 0.0 32, η2: 0.9828, 95% CI(-0.7838 to -0.5408), c: * *P*-value: 0.0 46, η2: 0.9946, 95% CI(-1.091 to -0.03779). d: η2: 0.95178, 95% CI(-0.5538 to -0.1033), e: η2: 0.9600, 95% CI(-0.9700 to -0.2270). The expression of *ACKR3* mRNA was normalized to that of the *β ACTIN *gene and analyzed via the delta deltaCT method. To assess the effects of hyperglycemia on *ACKR3* expression in endothelial cells, HUVECs were treated with 5 mM D-glucose (normal glucose, NG) or 25 mM D-glucose (high glucose, HG). (f) The *ACKR3* mRNA levels were increased at 24 and 48 h after hyperglycemic treatment in HUVECs. Data are mean ± SEM (n = 3) with One-way ANOVA, *P*-value: 0.015, ω^2^ = 0.951 [95% CI: 0.957- 0.992]. The ACKR3 protein (g & h) level was increased after 48h from hyperglycemic treatment in HUVECs. The reported values are the means of three independent replications of Western blot analysis. * *P*-value:0.024, η2: 0.9524, 95% CI(0. 2785 to 1.4620). * *P* < 0.05; ** *P* < 0.01

###  ACKR3 Regulates Several Cellular Pathways

 The KEEG and Harmonizome databases were used to predict the function of the *ACKR3* gene. The results showed that *ACKR*3 might be involved in insulin secretion, insulin resistance, inflammatory signaling pathways, and Transendothelial migration. Additionally, the *ACKR3* gene may be involved in vascular diseases and endothelial cell proliferation disorders (Supplementary File 2). Moreover, the JASPAR web showed that the ACKR3 promoter has a binding site for NFKB subtype (Supplementary File 1, Figure S2).

###  Knockdown of ACKR3 Attenuates Inflammation Under Hyperglycemic Conditions

 Previous studies have shown that hyperglycemiapromotes inflammation predominantly via increased activation of NF*-Κb.*^32^ Consistent with this, our result demonstrated that p65 protein expression was significantly increased by HG treatment. Following confirmation of ACKR3 knockdown at both mRNA and protein levels, we observed a significant decrease in the level of p65 protein (Figure 3). Additionally, we found that HG treatment enhanced the expression of *IL*-6 mRNA after 24 and 48 hours. However, increased levels of the IL-6 secretory protein were observed only after 48 h of HG treatment. The data revealed that the knockdown of *ACKR3* decreased IL-6 at both the mRNA and protein levels (Figure 4a and b).

**Figure 3:**
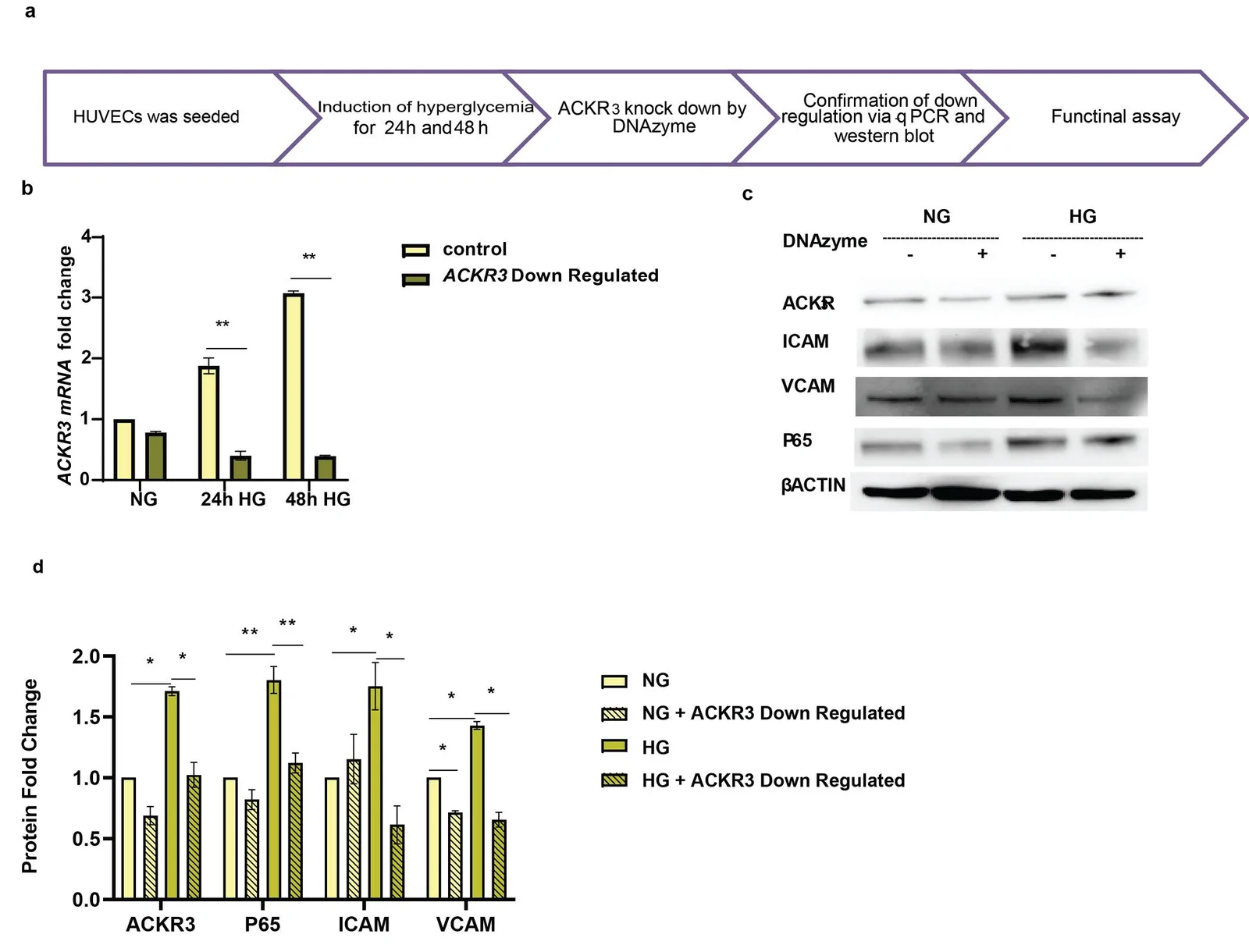
Response of ACKR3-downregulated HUVECs to hyperglycemic conditions.a: HUVECs were treated with NG or HG for 24 or 48 h, after which the cells were transfected with ACKR3 DNAzymes or scrambled DNAzymes to downregulation ACKR3 expression. ACKR3 expression was decreased after ACKR3 DNAzyme transfection at b: the expression of *ACKR3* mRNA, DNAzyme of ACKR3 decreased expression of *ACKR3* with *P*-value: 0.002. Data are mean ± SEM (n = 3) with One-way ANOVA, ω^2^ = 0.983 [95% CI: 0.957- 0.990]. The mRNA expression was normalized to the corresponding *β ACTIN* expression (n = 3 independent experiments) and was estimated via the 2^-∆∆Ct^ method. c&d: expression of protein levels. At the protein level, DNAzyme of ACKR3 decreased the level of ACKR3 protein with *P*-value: 0.027. the expression of p65, an important regulator of inflammation, was quantified via western blotting. Hyperglycemia increased P65 levels (*P*-value: 0.0075) and ACKR3 downregulation significantly reduced the level of p65 under NG and HG conditions (*P*-value:0.009). Additionally, ACKR3 downregulation significantly decreased ICAM and VCAM expression at the protein level under both conditions. ** *P* < 0.01. Protein intensity values were normalized to the relative β-actin value (n = 3 independent experiments). Densitometry plots reporting the fold change versus the control. Images report one representative experiment out of at least 3 independent experiments

**Figure 4:**
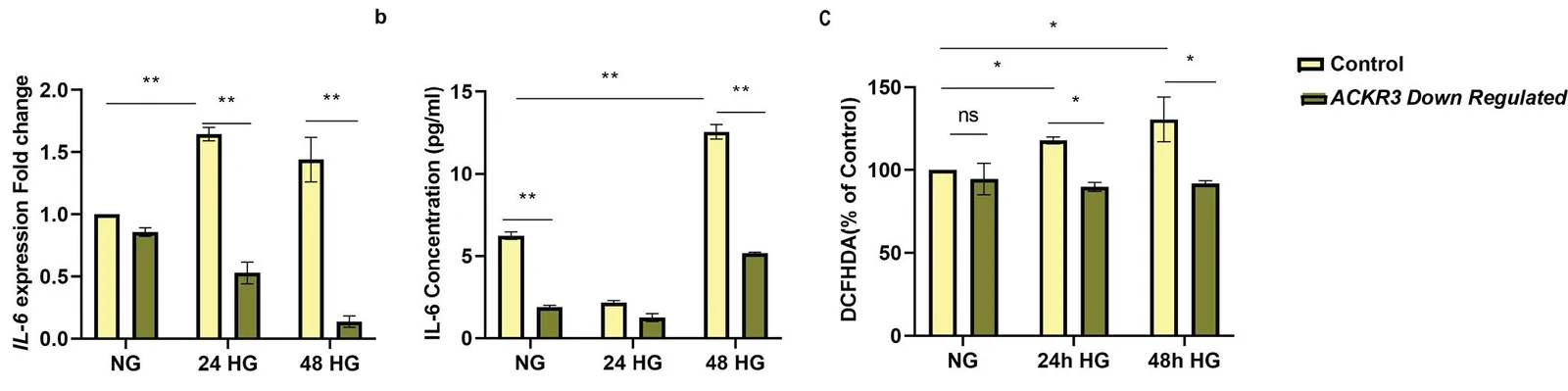
ACKR3 knockdown decreased inflammation under HG conditions. a: To elucidate the role of ACKR3 in inflammation progression, HUVECs were treated with NG or HG and then transfected with ACKR3 DNAzymes or scrambled DNAzymes. qPCR was performed to analyze the expression level of the *IL*-6 gene, and the resulting mRNA expression was normalized to the corresponding *β ACTIN* expression. Hyperglycemia increased the expression of *IL*-6 (*P*-value: 0.004), and ACKR3 downregulation decreased it (*P*-value: 0.009). Data are mean ± SEM (n = 3) with One-way ANOVA, ω^2^ = 0.78 [95% CI: 0.55 – 0.87] b: ELISA assay revealed that hyperglycemia enhanced IL-6 protein levels (*P*-value: 0.002). The downregulation of *ACKR3* decreased the IL-6 protein level (*P*-value: 0.0012). Data are mean ± SEM (n = 3) with One-way ANOVA,ω^2^ = 0.987 [95% CI: 0.970 – 0.994], 3 independent biological experiments within each group. c: Intracellular ROS production. To investigate the effect of ACKR3 downregulation on ROS generation, a DCFDAD assay was performed. The data are expressed as a percentage (%) of DCFH-DA fluorescence intensity relative to the control. A decreased level of ROS was observed after *ACKR3* downregulation (*P*-value: 0.021). Data are mean ± SEM (n = 3) with One-way ANOVA, ω^2^ = 0.69 [95% CI: 0.39 – 0.80], *P*-value of effect of HG: 0.010.* *P* < 0.05; ** *P* < 0.01. The results are presented as the average values from 3 independent biological experiments and two technical replicates

###  Knockdown of ACKR3 Decreased the Production of ROS Under Hyperglycemic Conditions

 Hyperglycemia is known to increase ROS production, which subsequently contributes to endothelial dysfunction and accelerates the development of vascular complications. Previous research has confirmed that high glucose in HUVECs increases intracellular ROS production.^33^ Our results confirmed that the level of intracellular ROS increased after HG induction (*P*-value: 0.01). Then, knockdown of *ACKR3* suppressed HG-mediated intercellular ROS production (*P*-value: 0.021), which likely indicates that the increased expression of *ACKR3* that occurs due to hyperglycemia may be effective in activating pathways related to ROS production (Figure 4c). Moreover, measurement of ROS production in mitochondrial fractions indicated that the level of ROS generation in mitochondria increased after hyperglycemia treatment, and downregulation of *ACKR3* decreased it (Supplementary File 1, Figure S3).

###  Downregulation of ACKR3 Increased the Activity of SOD and CAT

 Our data demonstrated that hyperglycemia significantly decreased the activity of SOD and CAT enzymes by about 30% (*P-value: 0.014) and 15% (*P-value: 0.010), respectively. *ACKR3* downregulation recovered the activity of SOD and CAT enzymes in hyperglycemic conditions (Supplementary File 1, Figure S3).

###  Knockdown of ACKR3 Inhibited HG-Mediated Apoptosis

 Apoptosis in endothelial cells is a critical mechanism contributing to vascular damage, leading to increased vascular permeability, inflammation, and leukocyte migration.^34^ To investigate the correlation of the *ACKR3 *downregulation with endothelial cell apoptosis under hyperglycemic conditions, the *ACKR3* gene was downregulated in HG and NG. Consistent with expectations, apoptosis significantly increased in HUVECs exposed to HG at 24h & 48h (*P*-value: 0.034 & 0.003), accompanied by an increased ratio of *BAX/BCL2* expression (*P*-value: 0.005). Notably, *ACKR3* knockdown decreased HG-induced increase in the expression of *BAX/BCL2* (*P*-value: 0.01) and reduced cell apoptosis (*P*-value: 0.004) (Figure 5). Our results suggest that the upregulation of *ACKR3* is associated with enhanced endothelial cell apoptosis under hyperglycemic stress.

**Figure 5:**
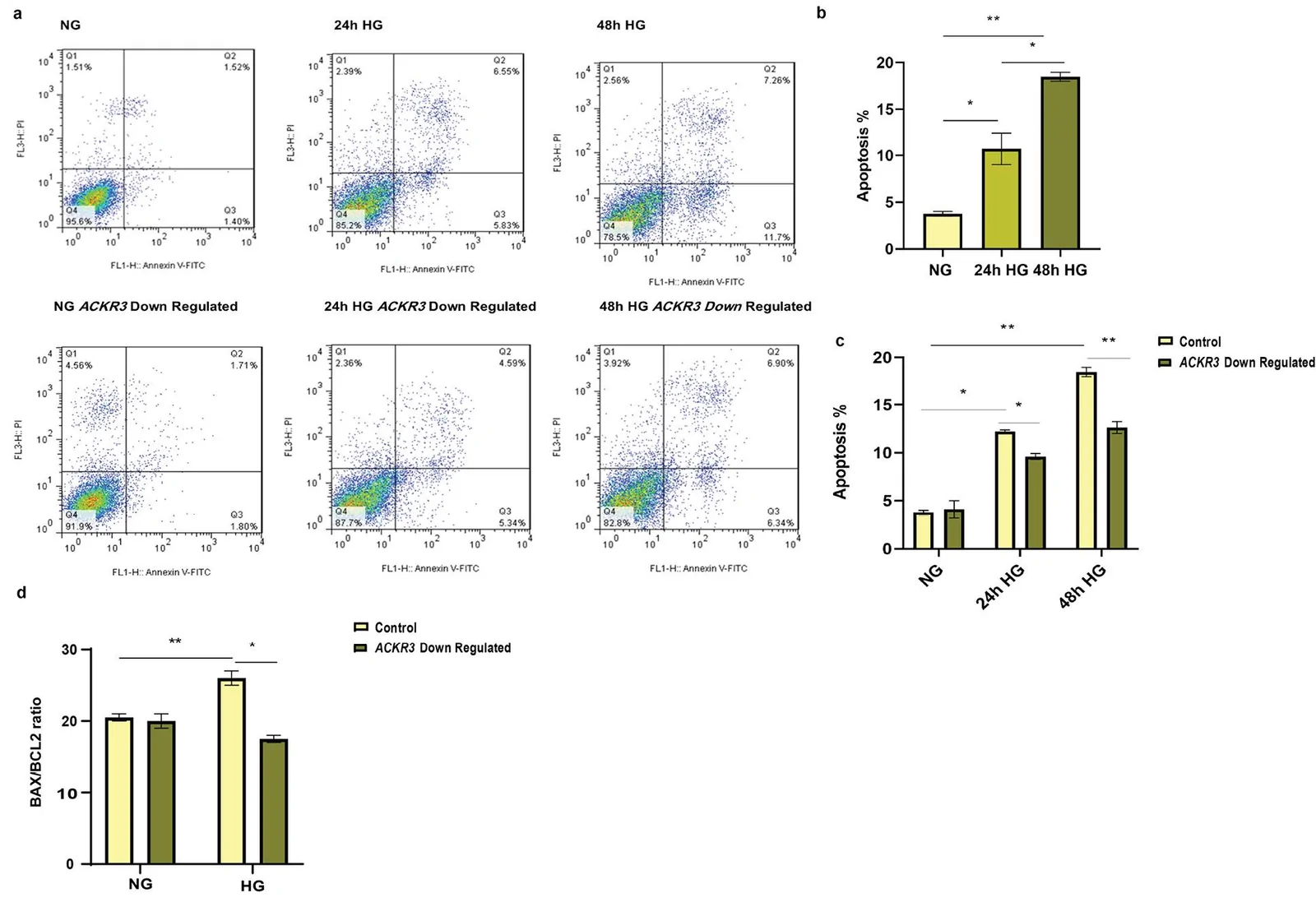
Knockdown of ACKR3 inhibits HG-mediated apoptosis. a: Flow cytometry histogram via Annexin V-propidium iodide (PI) staining. b: Annexin V-propidium iodide (PI) staining revealed that the rate of apoptosis increased in ECs after 24 and 48 h of HG treatment, with *P*-value 0.034 & 0.003. Data are mean ± SEM (n = 3) with One-way ANOVA, ω^2^ = 0.984 [95% CI: 0.931 – 0.993].c: ACKR3 downregulation reduced the percentage of apoptotic endothelial cells. Data are mean ± SEM (n = 3) with One-way ANOVA, ω^2^ = 0.982 [95% CI: 0.956 – 0.991], with *P*-value: 0.004. Additionally, the *BAX/BCL2* ratio was analyzed by qPCR, and mRNA expression was normalized to the corresponding *β ACTIN* expression. Hyperglycemia increased the levels of *BAX/BCL2 *mRNA (*P*-value: 0.005), and a decreased level of *BAX/BCL2 mRNA* was observed after ACKR3 knockdown with *P*-value: 0.01, data are mean ± SEM (n = 3). One-way ANOVA: ω^2^ = 0.927 [95% CI: 0.811 – 0.965]. For the flow cytometry panel, firstly, cells were gated based on FSC-A versus SSC-A to exclude cell debris. Subsequently, the target cell population was selected using specific markers PI and Annexin V. Finally, apoptosis was assessed by combining the cells in gates two and three. The results are presented as the average values from independent experimental samples (n = 3) within each group. * *P* < 0.05; ** *P* < 0.01

###  ACKR3 Downregulation Increased the Proliferation of HUVECs

 Previous studies have shown that hyperglycemic conditions inhibit endothelial cell proliferation. ^35^ To investigate the role of the ACKR3 in regulating endothelial cell proliferation under hyperglycemic stress, the *ACKR3* gene was downregulated in HUVECs treated with HG and NG. HUVECs’ proliferation was reduced in HG conditions (*P*-value: 0.045). So, *ACKR3* downregulation promoted the proliferation of HUVECs exposed to HG (*P*-value: 0.003) (Figure 6). These results suggest that *ACKR3* downregulation can alleviate the inhibitory effect of hyperglycemia on endothelial cell proliferation.

**Figure 6:**
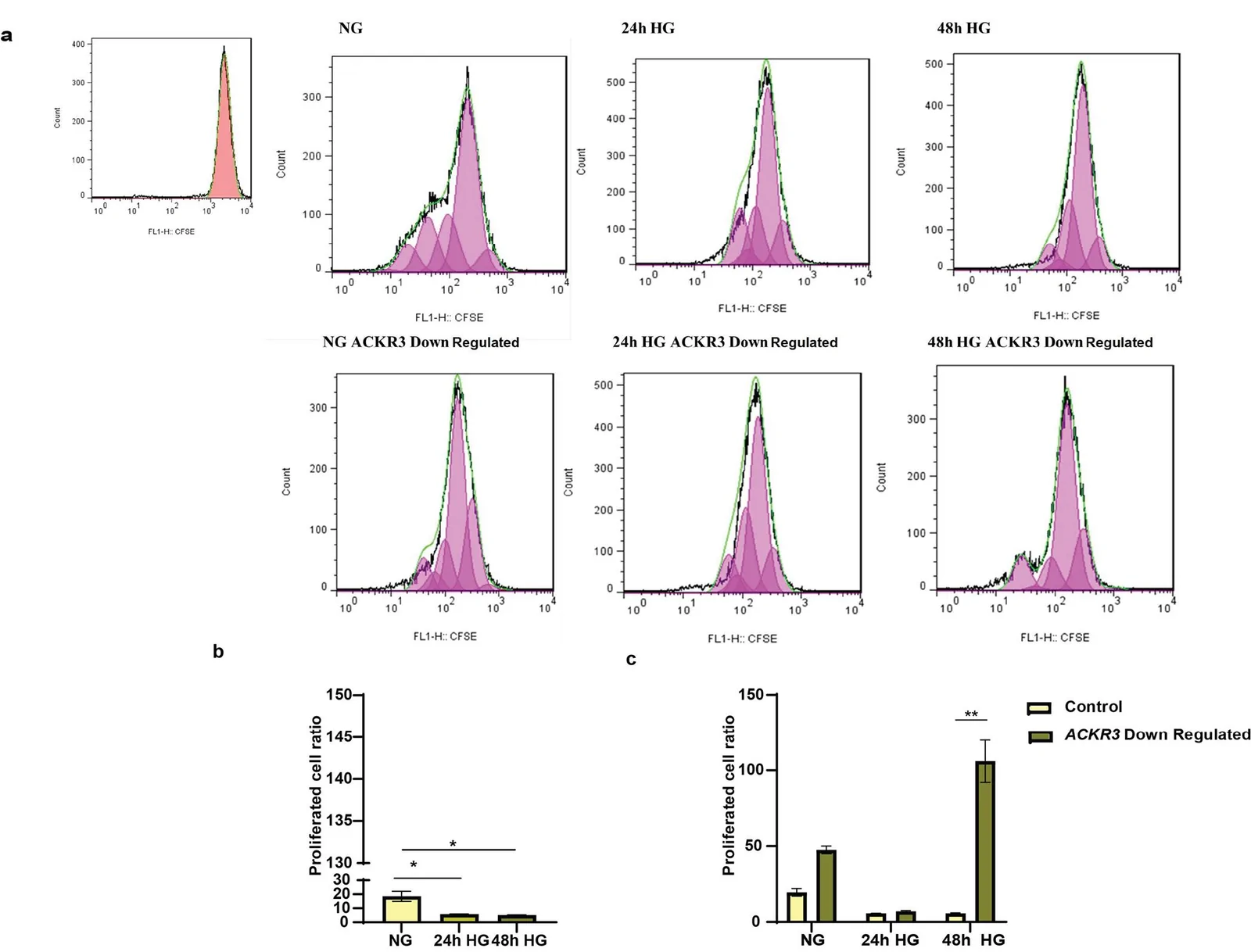
ACKR3 downregulation increased the proliferation of HUVECs. To elucidate the role of the ACKR3 in cell proliferation, HUVECs were treated with NG or HG for 24 h or 48 h and then transfected with ACKR3 DNAzymes or scrambled DNAzymes. a: A flow cytometry CSFE assay was performed to evaluate the impact of *ACKR3* on HUVEC cells. b: Data showing that cell proliferation decreased in ECs in response to HG treatment, with *P*-value: 0.045. (n = 3 independent experiments), One-way ANOVA: ω^2^ = 0.964 [95% CI: 0.872 – 0.983]. c: The data showed that the downregulation of ACKR3 promoted the proliferation of cells under HG conditions (*P*-value:0.003). The results are presented as the average values from independent experimental samples (n = 3) within each group. One-way ANOVA: ω^2^ = 0.978 [95% CI: 0.944 – 0.988]. * *P* < 0.05; *** *P* < 0.001

###  Knockdown of ACKR3 Decreased Cellular Senescence Under Hyperglycemic Conditions.

 Previous studies have shown that hyperglycemia exacerbates cellular senescence and hampers endothelial cell proliferation.^36^ the present study aims to elucidate the role of the *ACKR3* gene in regulating the senescence of endothelial cells under hyperglycemic conditions. Flow cytometry analysis revealed that under hyperglycemic conditions, cell cycle progression is arrested in the G1 phase, and genomic content in the S phase was reduced. Conversely, the downregulation of ACKR3 notably increased the number of endothelial cells that progressed through S phase. Additionally, X-gal staining revealed that senescence in HUVECs increased under HG conditions, whereas the downregulation of *ACKR3* expression mitigated senescence under HG conditions (Figure 7).

**Figure 7:**
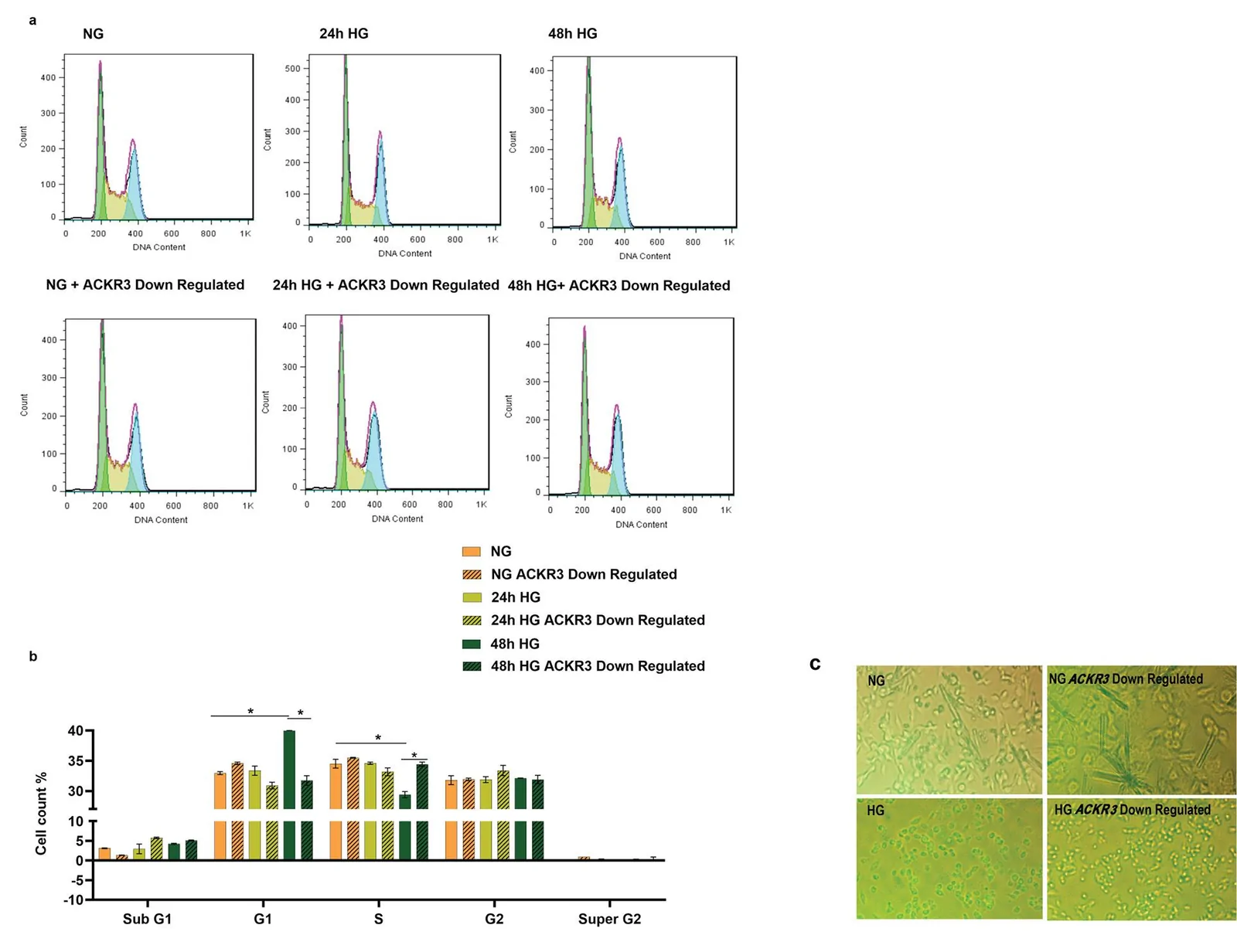
ACKR3 downregulation decreased EC senescence. To elucidate the impact of the *ACKR3* gene on cell senescence, HUVECs were treated with NG or HG for 24 h or 48 h and then transfected via ACKR3 DNAzymes or scrambled DNAzymes. a: PI staining was used for the cell cycle study. b: HUVEC cells showed an increased population in G1 in 48h HG condition(*P*-value: 0.018). Following *ACKR3* downregulation, the number of cells in the G1 phase decreased (*P*-value: 0.014). Data are mean ± SEM (n = 3), one-way ANOVA, ω^2^ = 0.936 [95% CI: 0.867 – 0.966]. Additionally, hyperglycemia decreased cells in S phase (*P*-value: 0.021), and *ACKR3* downregulation increased the percentage of ECs in the S phase at 48 h of HG treatment (*P*-value: 0.0175). Data are mean ± SEM (n = 3). One-way ANOVA: ω^2^ = 0.910 [95% CI: 0.818 – 0.949], *P*-value: 0.032. c: X-gal staining revealed that ACKR3 downregulation inhibited EC senescence under HG conditions. Cells with green–blue staining represent cellular senescence, magnification: 100x. The results are presented as the average values from three independent experimental samples within each group. * *P* < 0.05

###  Knockdown of ACKR3 Reduced Markers of Endothelial Dysfunction and Monocyte Adhesion

 Endothelial dysfunction, an early stage of atherosclerosis, is characterized by the overexpression of adhesion molecules, including ICAM and VCAM. Previous studies have indicated that hyperglycemia increases the expression level of ICAM and VCAM markers, thereby facilitating leukocyte recruitment to inflamed vascular sites.^37^ Therefore, to assess the impact of the *ACKR3* gene in regulating monocyte adhesion under hyperglycemia stress, we evaluated changes in ICAM and VCAM expression (Figure 3) as well as leukocyte attachment to HUVECs. As anticipated, HG treatment significantly increased ICAM and VCAM expression at both mRNA and protein levels. Remarkably, *ACKR3* downregulation significantly reduced the expression of these adhesion molecules under both NG and HG conditions. Correspondingly, monocyte adhesion decreased following *ACKR3* downregulation in HG conditions (Figure 8).

**Figure 8:**
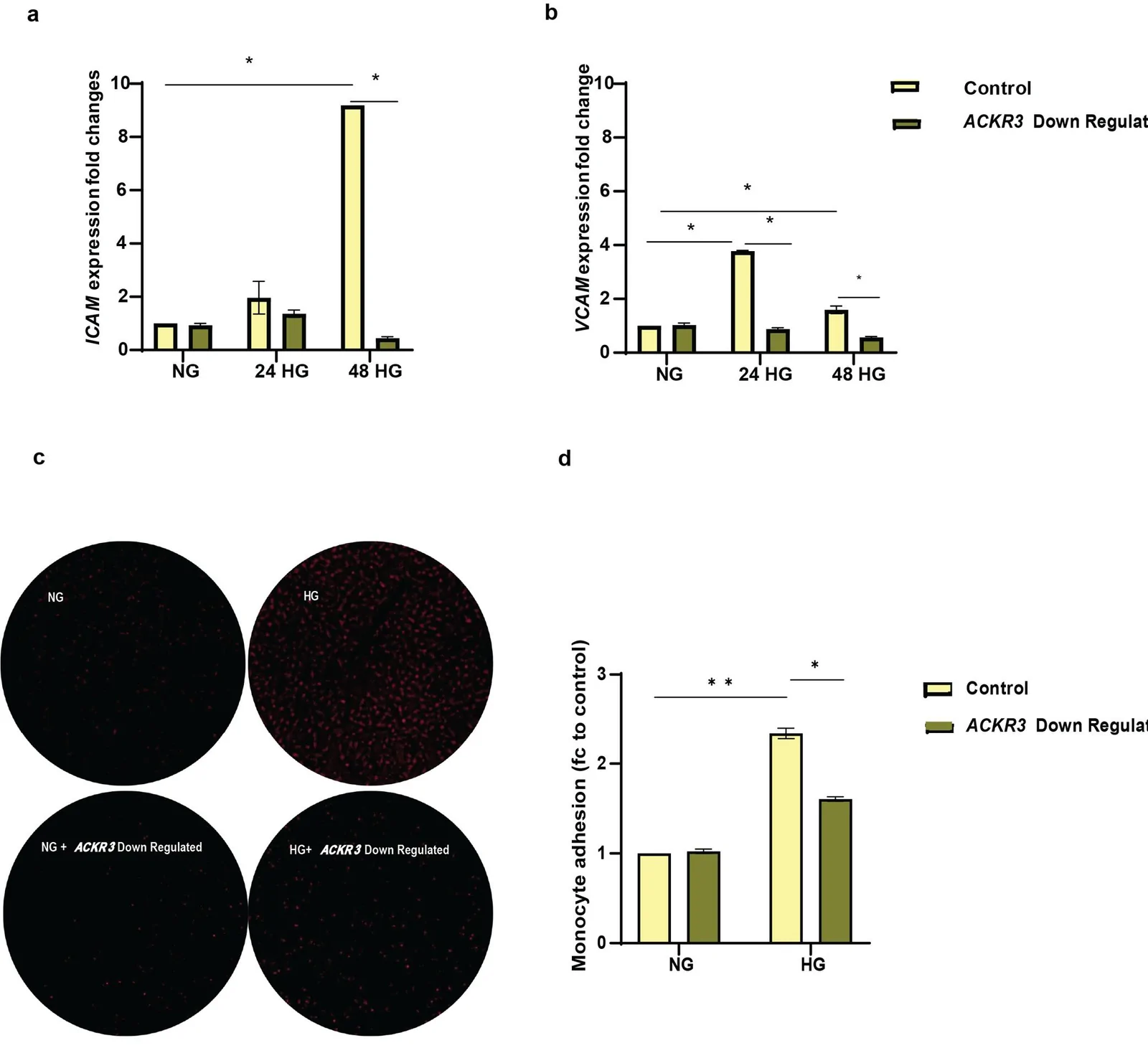
Knockdown of ACKR3 reduced monocyte adhesion. a: *ICAM* mRNA expression, data are mean ± SEM (n = 3). One-way ANOVA: ω^2^ = 0.962 [95% CI: 0.915 – 0.980] with *P*-value < 0.048. b: *VCAM* mRNA expression, Data are mean ± SEM (n = 3). One-way ANOVA: ω^2^ = 0.984 [95% CI: 0.960 – 0.993]. Hyperglycemia increased the expression of *VCAM* mRAN with a *P*-value: 0.04. and *ACKR3* downregulation decreased *VCAM* expression by *P*-value: 0.034. mRNA expression was assessed by qPCR, and the mRNA expression levels were normalized to the corresponding *β ACTIN* expression levels. c & d: Additionally, a monocyte adhesion assay revealed that hyperglycemia increased monocyte adhesion (*P*-value: 0.009) and *ACKR3* knockdown in ECs can reduce the number of THP-1 cells that adhere to HUVECs under HG conditions (*P*-value: 0.022). Images in d panel are microscopic fluorescence images. The small and bright spherical dots represent THP-1 cells that were pre-labeled with Red CMTPX dye, magnification: 100x Fluorescent light of adherent THP-1 to HUVECs was measured by excitation/emission (577 nm/ 602 nm) using fluorescence spectrophotometry. Data represented as fold change to control. All values are presented as the means of at least 3 independent experiments * *P* < 0.05; ** *P* < 0.01

## Discussion

 DM is a global disease with increasing incidence. Over time, 70% of diabetic patients are prone to various CVDs, particularly atherosclerosis.^2^ Endothelial dysfunction driven primarily by hyperglycemia and hyperlipidemia is an early event in the initiation and development of vascular inflammation in diabetic patients.^4^ Heightened inflammation in endothelial cells promotes cellular apoptosis and impairs their regenerative capacity.^33^ The activated endothelial cells secrete cytokines that facilitate the recruitment of monocytes, which play a pivotal function in the development of the inflammatory response in arteries during atherosclerosis.^10^

 GPCRs have an important impact on the progression of inflammatory disorders ^14^ and mediate cellular responses to various cytokines. Previous studies have reported that some GPCRs, such as GPR26, GPR39, and GPR22, are differentially expressed in both animal models of diabetes and diabetes patients.^13,14^ GWAS has revealed that certain genetic variants, such as single-nucleotide polymorphisms in GPCRs, increase susceptibility to diabetes, obesity, insulin resistance, and subsequent cardiovascular complications.^14^ These findings have led to increased interest in the potential of GPCRs as therapeutic targets for inflammatory disease treatment. Accordingly, we sought to elucidate the function of the ACKR3 GPCR in vascular complications related to diabetes.

 The *ACKR3* gene is located at 2q37, a genomic region implicated in diabetes susceptibility due to its proximity to the *CAPN10* gene, one of the first genes associated with type 2 diabetes. Previous studies have demonstrated that the ACKR3 GPCR exhibits a high affinity for cytokines such as ADM, CXCL11, and CXCL12.^38^ Notably, the circulating levels of these cytokines are significantly altered in both diabetes and CAD.^39–41^ Consequently, dysregulation in ACKR3 activation by these ligands may critically impact the initiation and progression of inflammatory responses associated with diabetes. Despite this potential, the regulatory function of ACKR3 in endothelial cells exposed to hyperglycemic stress remains unexplored.

 Our data revealed that *Ackr3* expression decreased in both ventricles and atria of diabetic rats. Interestingly, in contrast to the cardiac tissues, we observed an increase in *Ackr3* expression in the vasculature of diabetic rat models, suggesting a tissue-specific regulation of *Ackr3* in diabetes.

 Our data demonstrated that hyperglycemia significantly increased expression of ACKR3 at both the mRNA and protein levels. This upregulation was correlated with elevated levels of ROS production, increased expression of endothelial dysfunction markers, and enhanced endothelial cell apoptosis. Conversely, *ACKR3* knockdown decreased inflammation and the expression of endothelial dysfunction markers, such as ICAM and VCAM, resulting in decreased monocyte adhesion. These adhesion molecules facilitate monocytes’ binding to endothelial cells by interacting with α4β1 integrins on the surface of monocytes.^42^ Furthermore, it has been reported that decreased expression of ACKR3 in arterial endothelial cells leads to a reduction in the levels of ICAM and E-selectin via the AKT and MAPK pathways, potentially influencing vascular permeability.^43^ Additionally, ACKR3 may have a critical function in endothelial progenitor cell junctions.^44^ Endothelial cells after stimulation with TNF-α and IL-1β and transfected with ACKR3 siRNA exhibit significantly reduced adherence to HUVEC monolayers. Moreover, the *Ackr3* deficiency in endothelial cells reduced monocyte adhesion to vascular endothelium and subsequently decreased atherosclerosis development in ApoE-/- mice.^43^

 Our findings demonstrated that ACKR3 downregulation led to a reduction in the level of P65, a subunit of the NF-κB transcription factor complex. P65 is involved in inflammatory signaling and the transcription of ICAM and VCAM. Also, the reduced levels of NF-κB decreased IL-6 production, which has been found to increase during hyperglycemia.^45^ Collectively, these results indicated that downregulation of ACKR3 decreased the inflammatory phenotype by suppressing P65 signaling under hyperglycemic stress. A previous study indicated that ACKR3 downregulation by siRNA resulted in a decrease in the level of P65 phosphorylation in endothelial cells.^43^ Similarly, inhibition of ACKR3 reduces NF-κB phosphorylation in inflammatory disease models, such as acute pulmonary inflammation and acute peritonitis.^46^ Promoter analysis of *ACKR3* via the JASPAR web server identified a putative NF-κB binding site. This suggests that NF-κB may directly regulate *ACKR3* transcription. These points may point to a potential positive feedback loop whereby reduced NF-κB levels following ACKR3 downregulation further suppress ACKR3 expression.

 Hyperglycemia promotes ROS generation, followed by an increase in cell apoptosis. Our data indicate that *ACKR3* knockdown reduced ROS production and cell apoptosis caused by glucose stress. In fact, downregulation of ACKR3 in hyperglycemic conditions may suppress excessive production of ROS, potentially by regulating the activity of Antioxidant enzymes like Catalase (CAT) or Superoxide dismutase (SOD). This process induced endothelial cell maintenance by decreasing cellular senescence and apoptosis. Prior studies have shown that ACKR3 may play a role in regulating cell death and survival.^47^ However, in contrast to our findings, another study demonstrated that ACKR3 plays a role in the preservation and survival of monocytes and increases binding to platelets.^48^

 The activation of ACKR3 in various pathological conditions has been associated with diverse roles in cardiovascular disease, with both aggravating and protective effects depending on the tissue type, cell type, and nature of acute or chronic inflammation.^38^ A precise understanding of the underlying mechanism by which ACKR3 regulates diabetic cardiovascular complications could facilitate the identification of a suitable therapeutic target in the future.

 In fact, this study has some limitations, such as RNAseq analysis of other data with a larger sample size. The analysis of gene expression primarily in circulating endothelial cells. Future studies should examine other vascular-related cell types, such as monocyte and lymphocyte cells, under hyperglycemic conditions, which can also provide more information. The use of inhibitors related to the inflammatory and ROS pathways can also reduce the limitations of this study and provide strong confirmation of the role of this gene in regulating inflammatory pathways. NF-κB activity assays or promoter binding assays can reveal the regulatory mechanism of the *ACKR3* gene. Additionally, the use of female diabetic animal models can reveal the effects of various hormones on ACKR3 expression during diabetes and provide a broader understanding of ACKR3 functions. Additionally, the use of rescue experiments via *ACKR3* overexpression or an ACKR3 agonist can provide strong validation of the roles of ACKR3. Finally, the use of ACKR3 conditional knockout diabetic mice or ACKR3 siRNA confirmed the beneficial effect of ACKR3 knockdown on reducing diabetic complications.

## Conclusion

 In toto, this is the first study to demonstrate the impact of the *ACKR3* gene in endothelial cells under high-glucose stress. Our data demonstrated that hyperglycemia increased ACKR3 expression in HUVECs, correlating with increased endothelial cell dysfunction (oxidative stress, inflammation, and apoptosis). Nevertheless, further experimental validation is warranted to confirm the role of ACKR3 in diabetic conditions. We identified the potential role of targeting the *ACKR3* gene in HUVECs under hyperglycemia-mediated stress to mitigate the adverse effects of hyperglycemia.

## Competing Interests

 The authors declare no competing interests.

## Consent to Publish

 Not applicable.

## Data Availability

 Data is provided within the manuscript or Supplementary files (1 and 2), and any additional data related to this study are available from the corresponding author upon reasonable request.

 Also, the datasets extracted and analyzed during the current study are available in the GEO (https://www.ncbi.nlm.nih.gov/geo/) repository, with the accession numbers GSE77108, and GSE267930.

## Ethical Approval

 All experiments were conducted in accordance with the Guide for the Care and Use of Laboratory Animals and approved by the “Ethics Committee of Tarbiat Modares University” (IR.MODARES.REC.1399.192).

## Supplementary File

Supplementary file 1 contain Table S1-S3 and Figures S1-S3.

Supplementary file 2. Gene ontology and pathway
