# Supplementary material for: ACKR3 Upregulation Under Hyperglycemia Associates with Endothelial Dysfunction
Source: Adv Pharm Bull. 2026 May 10;16(3):617–30. doi: 10.34172/apb.46517 (PMC13745751; doi:10.34172/apb.46517)
Supplement: Supplementary file 1 [file apb-16-617-s001.pdf]

Adv Pharm Bull 2026;16(3):617-S1
doi:10.34172/apb.46517
https://apb.tbzmed.ac.ir

Table S1: RNA-seq datasets

**GEO data sets**		
**GSE77108**	Healthy (GSM2044419, GSM2044420, GSM2044421)	Diabetic patients (GSM2044434, GSM2044435, GSM2044436)
**GSE267930**	HUVEC control (GSM8281734, GSM8281735, GSM8281736)	HUVEC induced dysfunction (GSM8281740, GSM8281741, GSM8281742)

Table S2. Sequence of primers were used in this study to investigate gene expression changes via the qPCR.

**Gene**	**Transcript**	**Primer Name**	**Primer Sequence (5'→3')**	**Amplicon length(bp)**
ACKR3	NM_020311.3	Forward	ATTGCCGACCTGTGGGTTGT	325
		Reverse	AGTAGGTCTCATTGTTGGACGC	
ACTB	NM_001101.5	Forward	ACCCAGCACAATGAAGATCAAGA	175
		Reverse	ATAGTCCGCCTAGAAGCATTTG	
ICAM2	NM_001099789.2	Forward	AACTGCAGCACCACCTGTAA	266
		Reverse	CTGCACTCAATGGTGAAGGA	
VCAM1	NM_001078.4	Forward	GGGAACGAACACTCTTACCT	240
		Reverse	ATCCTCCAGAAATTCCTGACT	
IL6	NM_000600.5	Forward	CCAATCTGGATTCAATGAGGAG	200
		Reverse	GGTCAGGGGTGGTTATTGCATC	
BAX	NM_138761.4	Forward	GGAGCTGCAGAGGATGATTGCC	316
		Reverse	TCCCGCCACAAAGATGGTCACG	
BCL2	NM_000633.3	Forward	GATACTGAGTAAATCCATGCAC	356
		Reverse	AGTGTTGCAGAATATCAGCCAC	
Ackr3	NM_053352.2	Forward	TCCATCACCTACTTCACCAGCA	297
		Reverse	ACCGGATGCTGACATGGCTCTG	
Actb	NM_031144.3	Forward	CCTCTATGCCAACACAGTG	275

	Reverse	ACGCAGCTCAGTAACAGTCC

Table S3. List of used antibodies for Western blot (WB) analysis

Antigen	Supplier	Clone /Cat. no.	Concentration
NF-κB (p65)	Cell Signaling Technology		1:1000
β-actin	Santa Cruz Biotechnology	2418	1:4000
ACKR3	Abcam		1:500
ICAM	Santa Cruz Biotechnology	sc-8439	1:1000
VCAM	Abcam	EPR5047	1:1000
Goat anti-rabbit IgG (HRP)	Cell Signaling Technology	7074	1:2000

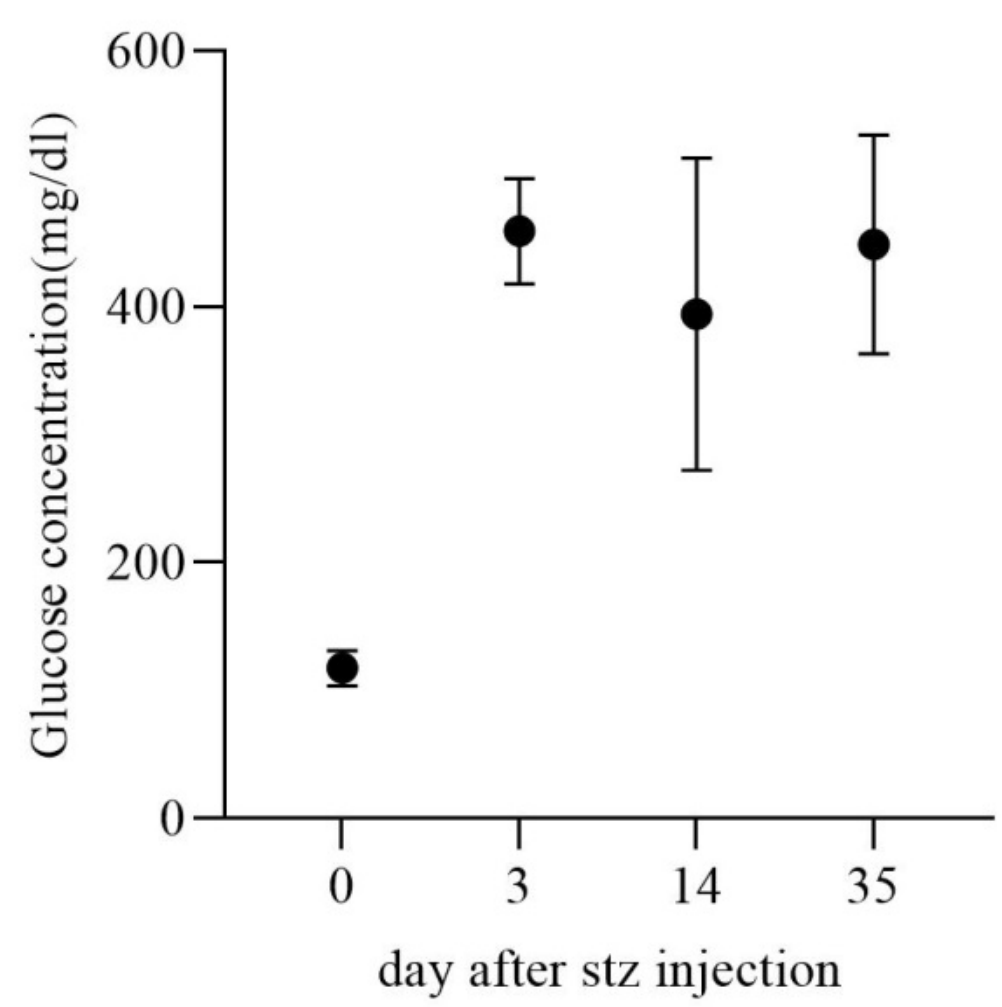

Figure S1: The blood glucose levels of the STZ diabetic rats were determined during the experiment, none of them showed the return of blood glucose levels.

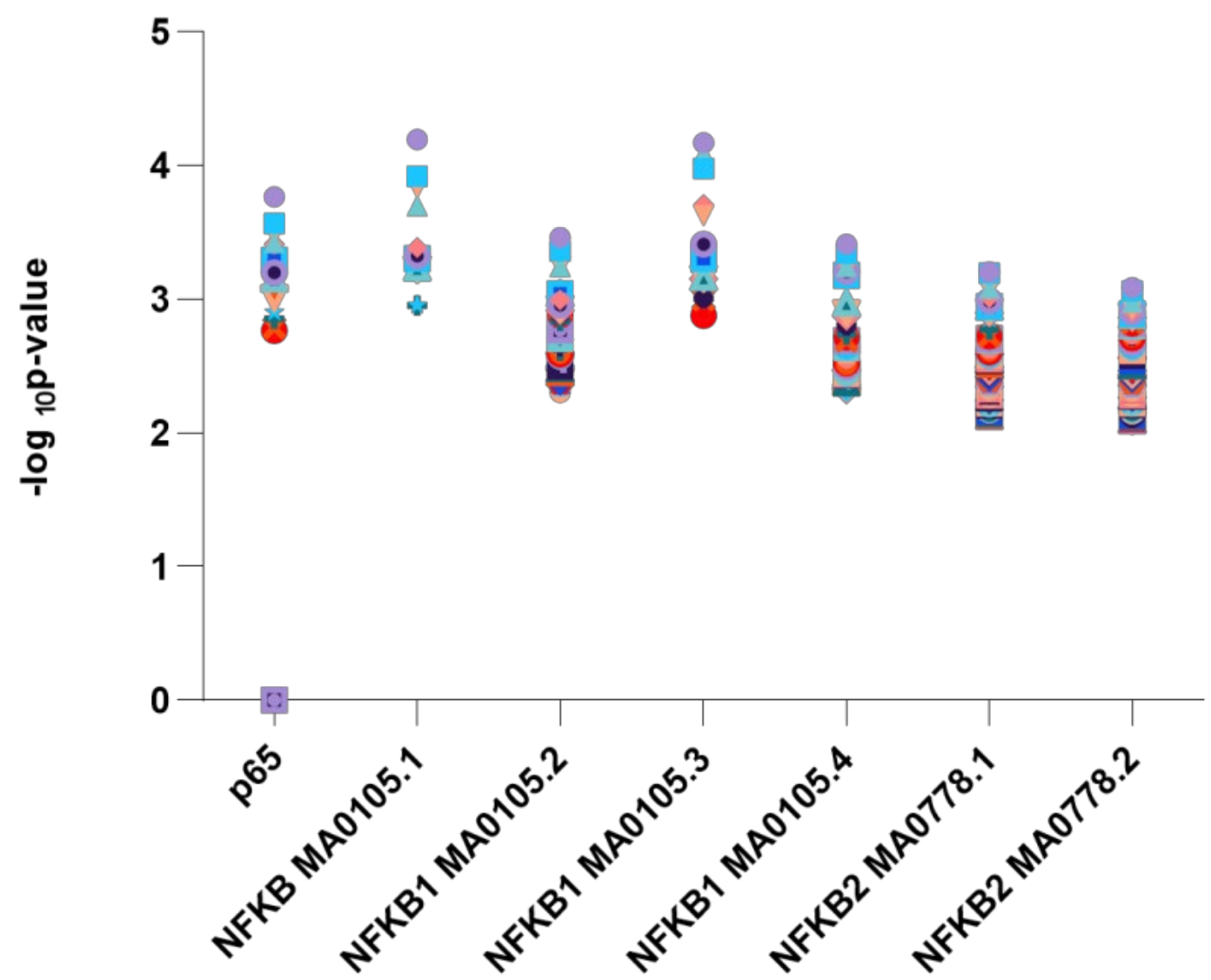

Figure S2: JASPAR web showed that the ACKR3 promoter has a binding site for NFKB subtype

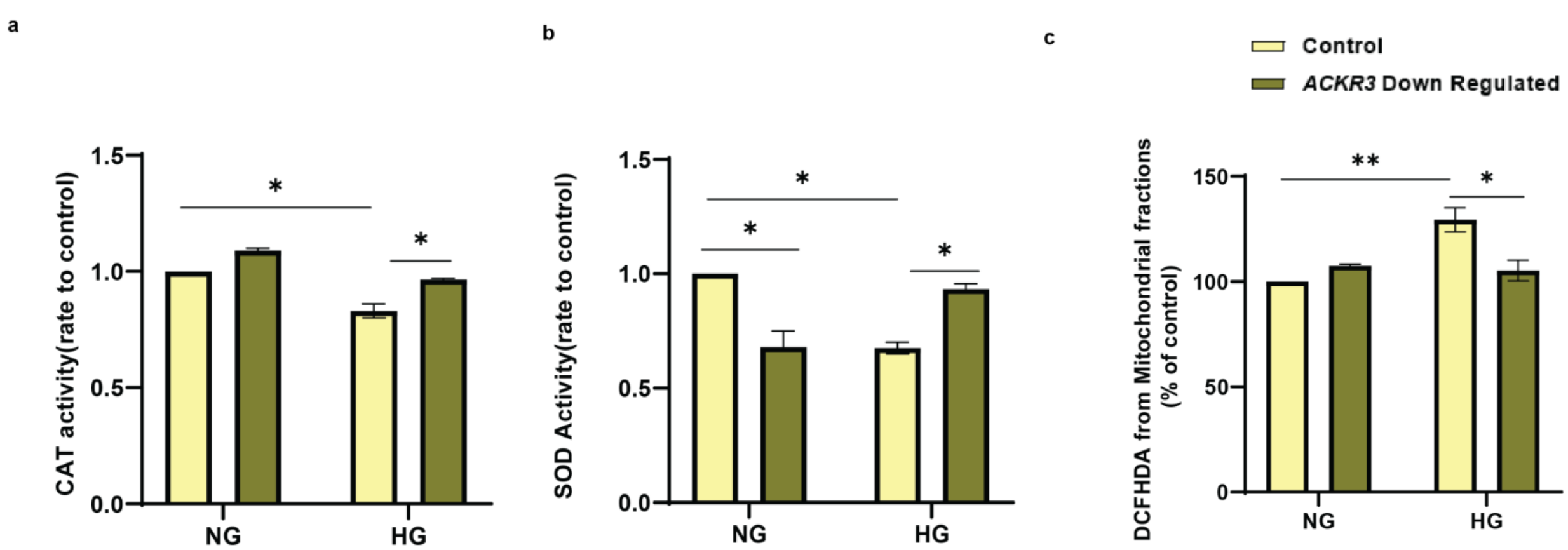

Figure S3: ACKR3 downregulation decreased mitochondrial ROS, which may be due to the recovery of antioxidant enzyme activity. a: CAT activity, Data are mean ± SEM (n=3) with One-way ANOVA, ω^2 = 0.972 [95% CI: 0.395 -0.897]. Hyperglycemia decreased CAT activity with p-value: 0.01, and ACKR3 downregulation increased CAT activity with p-value: 0.014. b: SOD activity, Hyperglycemia decreased SOD activity with p-value: 0.014, and ACKR3 downregulation increased SOD activity with p-value: 0.031, data are mean ± SEM (n=3) with One-way ANOVA, ω^2 = 0.990 [95% CI: 0.988 -0.898]. c: DCFHDA Measurement in Mitochondrial Fractions represents the rate of ROS production in Mitochondria. Data are mean ± SEM (n=3) with One-way ANOVA, ω^2 = 0.795 [95% CI: 0.395 -0.898]. Increased mitochondrial ROS by hyperglycemia, with p-value: 0.003, and ACKR3 downregulation decreased this level with p-value: 0.019.
